# Scurvy in the Modern Era: Revisiting a Forgotten Diagnosis in a Child With Chronic Recurrent Multifocal Osteomyelitis

**DOI:** 10.7759/cureus.91964

**Published:** 2025-09-10

**Authors:** Rita Abou Zeid, Jessica Nicolas, Ghassan Dbaibo, Mira Merashli

**Affiliations:** 1 Rheumatology, American University of Beirut Medical Center, Beirut, LBN; 2 Pediatrics, Faculty of Medicine, American University of Beirut Medical Center, Beirut, LBN

**Keywords:** bone pain in children, case report, chronic recurrent multifocal osteomyelitis (crmo), scurvy, vitamin c deficiency

## Abstract

Scurvy, an ancient disease caused by severe vitamin C deficiency, continues to resurface in modern settings, especially among patients with selective eating habits due to developmental disorders, feeding difficulties, or low socioeconomic status. This report describes the case of a three-year-old child who presented with five weeks’ history of multisystemic symptoms, such as systemic (fever, generalized edema), musculoskeletal (bone pain), dermatological (petechiae), and mucosal (gingivitis), leading to a diagnostic dilemma. The patient underwent extensive workups, including biopsies and infectious and autoimmune tests, all of which were negative. Clinical and radiological findings initially suggested chronic recurrent multifocal osteomyelitis (CRMO), which is a rare diagnosis of exclusion, frequently considered in children with unexplained multifocal bone lesions, and can easily lead to misdiagnosis. The turning point came from the child’s dietary history, primarily consisting of rice, raising suspicion for nutritional deficiency. Micronutrient testing revealed a severely low vitamin C level, confirming the diagnosis of scurvy. Oral supplementation resulted in the complete resolution of his gingivitis, petechiae, and edema within one week. Scurvy is often overlooked in modern practice despite its simple diagnosis and treatment with ascorbic acid. This case highlights the diagnostic challenge posed by the overlap of symptoms (bone pain) and radiological findings between scurvy and CRMO. It also emphasizes the importance of detailed dietary history and recognition of nutritional deficiencies in the differential diagnosis in children with multisystem involvement.

## Introduction

Scurvy, an ancient disease caused by severe vitamin C deficiency, is increasingly recognized in modern settings. It occurs in elderly, institutionalized individuals, and those from low socioeconomic background where variety in diet is restricted. It is also seen in children with complex medical conditions such as neurodevelopmental disorders, genetic syndromes, food allergies, coeliac disease, or eating disorders [1]. Scurvy can present with dermatological issues (petechiae, ecchymoses), gingival symptoms (swollen, bleeding gums), and musculoskeletal problems (bone pain, subperiosteal hemorrhages) [1]. Its symptoms may mimic serious conditions such as hematological malignancies, vasculitis, osteoarticular infections, and chronic recurrent multifocal osteomyelitis (CRMO) [2].

CRMO is an autoinflammatory disease and a diagnosis of exclusion in children presenting with unexplained recurrent multifocal bone pain and sterile inflammatory lesions [2]. Both CRMO and scurvy are rare diseases and can be easily overlooked if the treating physician is unfamiliar with them [2]. The overlapping clinical and radiological findings between CRMO and scurvy can delay diagnosis and appropriate treatment, thereby increasing morbidity [3].

We report an intriguing case of a three-year-old child who presented with a myriad of multiple-organ symptoms including fever, bone and joint pain, generalized edema, petechial rash, and gingivitis. This complex presentation and the poor dietary history taken initially masked the underlying nutritional etiology, prompting extensive and invasive diagnostic testing.

Scurvy has a simple diagnostic test and straightforward treatment. Yet it is often overlooked in modern medical practice as it is uncommon, typically seen in patients with restricted vitamin C intake, and mimics many pediatric diseases. This case underscores the importance of taking a detailed dietary history in all pediatric populations and considering nutritional deficiencies in the differential diagnosis, particularly when faced with complex and multi-system presentations [4].

## Case presentation

**Figure 1:**
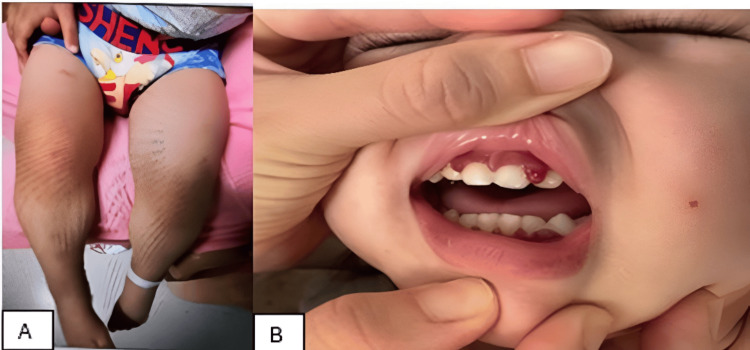
Left lower-limb edema (A). Gingival hypertrophy (B).

A three-year-old boy with a history of recurrent tonsillitis and normal developmental milestones presented with a five-week history of fever, tonsillitis, and inability to walk, initially manifesting as left-sided limping. On admission, his weight was 14 kg or 31 lb (reference range: 12-16 kg or 26-35 lb), corresponding to the 50th percentile for age. His height was 95 cm or 37.4 in (reference range: 90-100 cm or 35.4-39.4 in), corresponding to the 50th percentile for age. On physical examination, the child was hypotonic with brisk deep tendon reflexes and normal motor power. Prominent clinical findings included severe leg pain with left leg edema, petechiae on his back, and gingival hyperplasia with bleeding from his gum (Figures 1A, 1B). These features were striking and key diagnostic clues.

**Figure 2:**
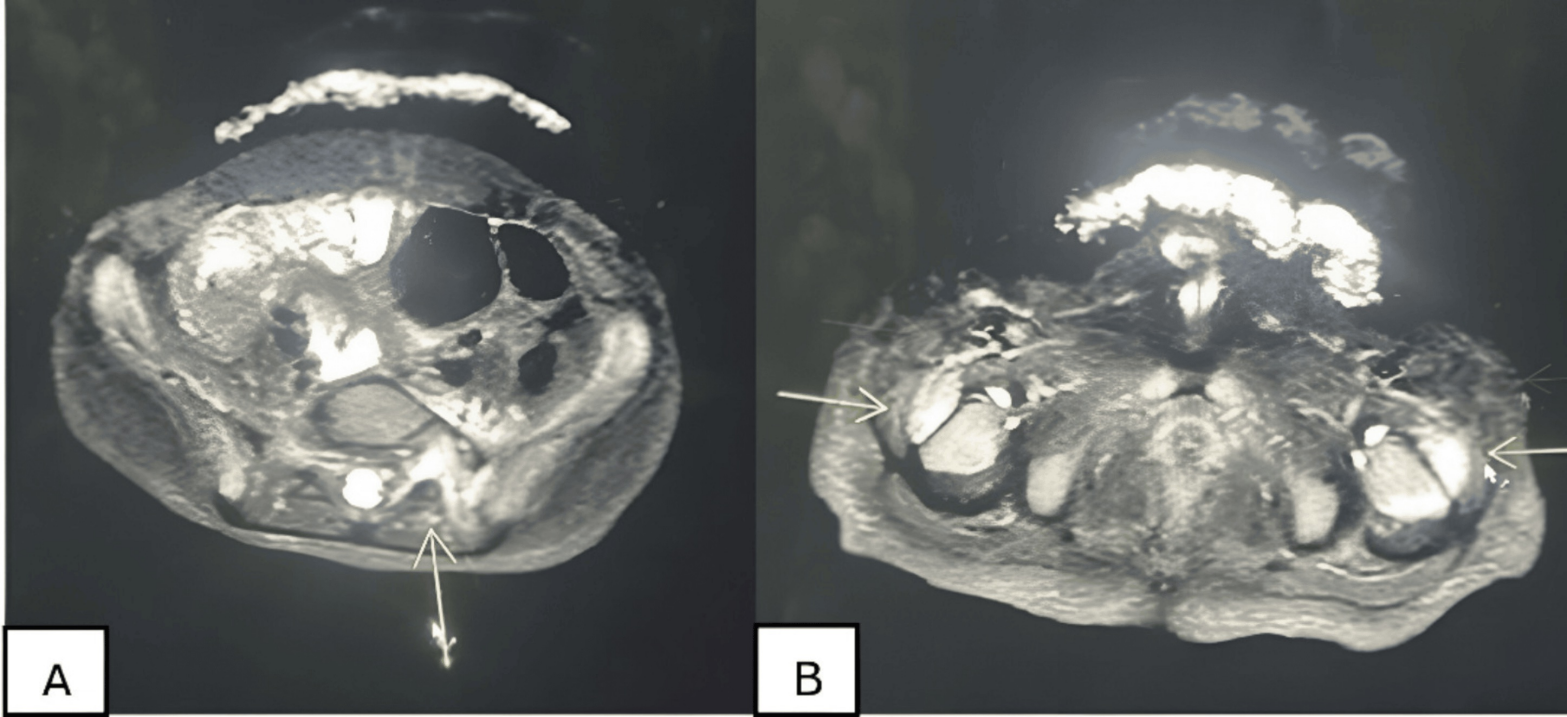
Full-body MRI showing septic arthritis of the left aspect of the L5 posterior elements (A) and iliac bones (B) with osteomyelitis (yellow arrows).

Laboratory results showed normal blood counts, as well as normal liver and kidney function. Inflammatory markers were slightly elevated with an erythrocyte sedimentation rate of 19 mm/hour (reference range: 0-15 mm/hour) and C-reactive protein of 4 mg/dL (reference range: 0.0- 2.5 mg/L). Full-body magnetic resonance imaging (MRI) revealed multifocal infectious/inflammatory changes in the femoral heads bilaterally and in multiple anterior and posterior ribs, with additional findings of pseudoarthrosis and suspected septic arthritis and osteomyelitis involving the fifth lumbar vertebra (L5) and the left iliac bone (Figures 2A, 2B). Differential diagnoses included multifocal osteomyelitis with septic arthritis, CRMO, and malignancy.

**Figure 3:**
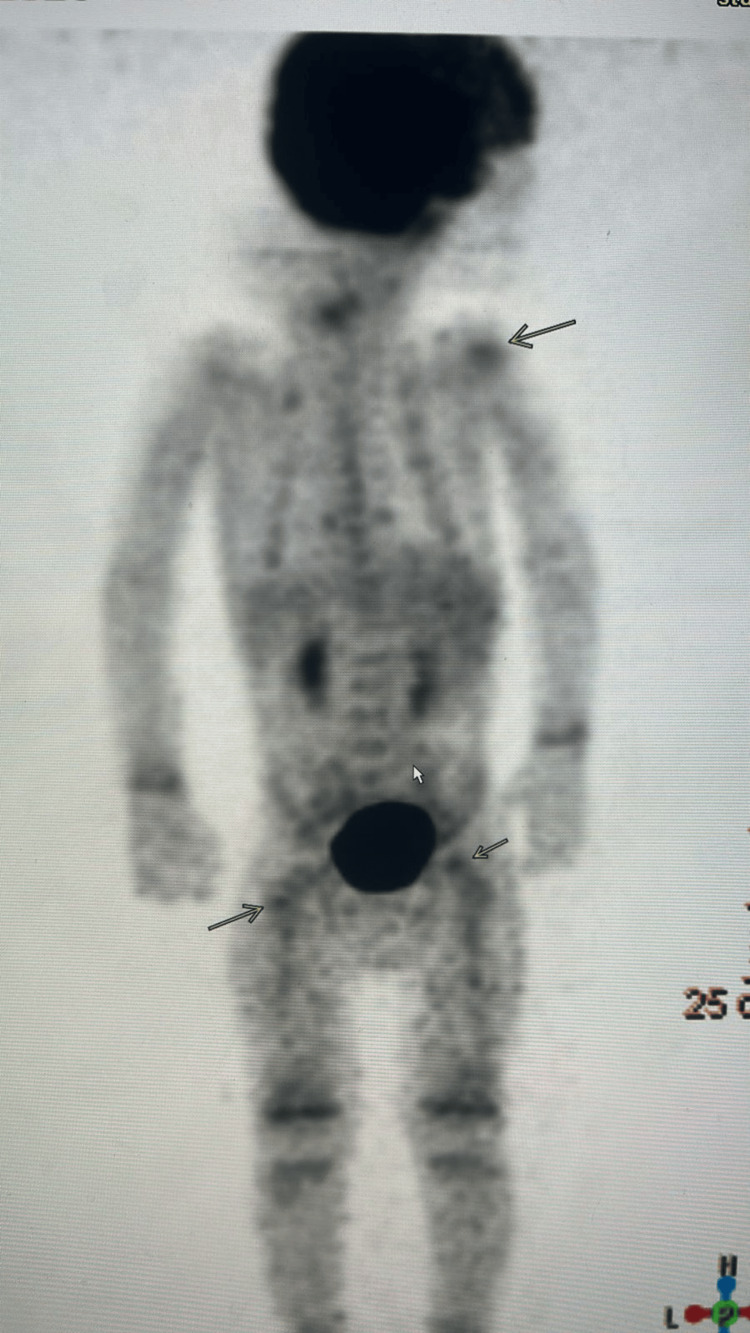
An 18-FDG PET scan showing faint uptake in the right humeral head and right femoral head (black arrows). 18-FDG PET, 18-fluorodeoxyglucose positron emission tomography

The child underwent extensive investigations, all of which were negative. The infectious workup included blood cultures, purified protein derivative (PPD), bone biopsies with cultures, and brucella titers, while the autoimmune evaluation consisted of rheumatoid factor (RF), antineutrophil cytoplasmic antibodies (ANCA), antinuclear antibody (ANA), immunoglobulins including IgG subclasses, complement levels (C3, C4), and serum protein electrophoresis. In addition, invasive biopsies were performed from the fifth vertebral body, skin (petechial lesions), and bone marrow. These repeatedly negative results significantly delayed the diagnosis. A total-body positron emission tomography (PET) scan was subsequently performed, which confirmed the MRI findings and ruled out malignancy (Figure 3).

**Figure 4:**
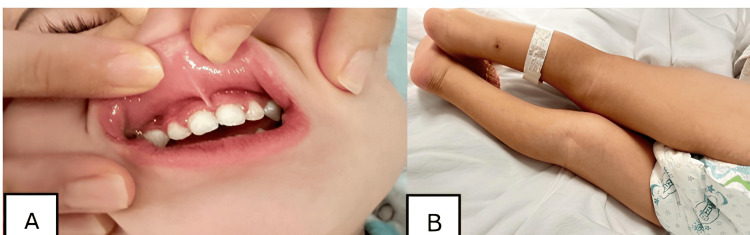
Complete resolution of gingivitis (A) and edema (B) after vitamin C intake.

Despite being from a wealthy family with no limitation in access to food, a detailed dietary history revealed that our patient was a picky eater, mainly relying on rice. This raised suspicion for nutritional deficiency. Micronutrient testing showed severely reduced vitamin C (<0.1 mg/dL) (reference range is 0.6-2 mg/dL), while other micronutrient levels (vitamin B, K, A) were slightly reduced but not clinically significant, confirming scurvy. Initiation of oral ascorbic acid supplementation was started at 500 mg daily. Although higher than the typical pediatric replacement dose (100-300 mg), this regimen was chosen to ensure rapid replenishment given the severity of presentation. Clinical improvement was dramatic, with complete resolution of gingival hyperplasia and edema within one week (Figures 4A, 4B).

Table 1 highlights the timeline of progression of the case from initial symptoms to the final diagnosis.

**Table 1:** Case timeline highlighting the progression of key events from initial symptoms to the final diagnosis and therapeutic response NSAID, nonsteroidal anti-inflammatory drug

Time point	Event
Week -5	Onset of symptoms: fever, tonsillitis, and left-sided limping progressing to inability to walk.
Weeks -5 to -1	Treatment with amoxicillin, NSAIDs, and steroids (4 weeks) without improvement.
Week 0	Presentation to the hospital with fever, severe leg pain, edema, petechiae, and gingival hyperplasia. Full-body magnetic resonance imaging (MRI) revealed multifocal infectious/inflammatory changes in the femoral heads bilaterally and in multiple ribs, with additional findings of pseudoarthrosis and suspected septic arthritis and osteomyelitis involving the fifth lumbar vertebra (L5) and the left iliac bone. Initial differential diagnoses included multifocal osteomyelitis, CRMO, and malignancy.
Week 1	Empiric treatment with antibiotics (cefepime + vancomycin) and corticosteroids.
Weeks 2–8	Persistence of symptoms despite treatment. Bone marrow aspirate and biopsy were within normal. Skin biopsy of back lesion showed hypersensitivity reaction with lymphohistiocytic infiltrates. PET-CT showed faint FDG uptake in bones consistent with MRI lesions.
Week 8	Confirmed diagnosis of scurvy based on vitamin C level <0.1 mg/dL.
Week 9	Initiation of oral ascorbic acid (500 mg daily).
Week 10	Marked clinical improvement: resolution of gingival hyperplasia, leg edema, and pain.

## Discussion

Diagnosing this case was challenging due to significant clinical and radiologic overlap between scurvy and CRMO, particularly in a normally growing child with no apparent risk factors.

CRMO was initially suspected because the child presented with recurrent bone pain, multifocal lesions on MRI, and persistently negative infectious workups - all consistent with an inflammatory bone disease [5,6]. CRMO is a diagnosis of exclusion, usually recurrent and often affecting the bones symmetrically. The typical imaging findings of CRMO include lytic and sclerotic lesions in the metaphysis of long bones and the medial clavicles. It can also affect the vertebral bodies, pelvis, ribs, and mandible, which closely mimicked the presentation of the child in our case [7,8].

Although CRMO was the primary diagnosis, several important clinical parameters were inconsistent with the classically described presentations of CRMO. First, CRMO typically affects children aged 7 to 12 years [5]. The child here was just three years old. Second, among the important features in this case were gingival hyperplasia and petechial rash, which are not typical of CRMO but characteristic of vitamin C deficiency [9]. In scurvy, petechiae arises due to vascular fragility caused by defective collagen synthesis [9,10]. This kind of rash is not characteristic of CRMO, as it does not have skin manifestation such as petechiae. Instead, pustular lesions on palms and soles, which are not present in this case, are characteristic [8].

Third, inflammatory markers were only mildly elevated, whereas higher levels are more common in CRMO [6]. Although osteomyelitis was observed on MRI, these findings may well be due to bone fragility with defective collagen synthesis in scurvy [11,12].

Moreover, the child's poor feeding habits, based mostly on eating rice, raised the possibility of nutritional deficiency, and the low vitamin C level confirmed the diagnosis. Thus, scurvy provided a more coherent explanation of the clinical, radiological, and laboratory findings.

Symptoms of scurvy often occur when the plasma concentration of ascorbic acid drops below 0.2 mg/dL (reference range is 0.6-2 mg/dL) [1]. However, the sensitivity and specificity of this diagnostic measure can vary after recent dietary intake [3]. Finally, the dramatic resolution of gingival changes, petechiae, and edema within one week of vitamin C supplementation confirmed the diagnosis of scurvy. Infants and children are usually treated with vitamin C 100-300 mg daily and adults 500-1,000 mg daily for one month or until full recovery of clinical signs and symptoms occurs [13]. Our patient received 500 mg of oral vitamin C daily to ensure rapid repletion in the context of severe symptomatic disease.

Table 2 provides a comprehensive comparison of scurvy and CRMO, highlighting major similarities and differences between the two diseases, regarding age of presentation, symptoms, imaging, laboratory findings, and treatment response. This practical framework demonstrates how gingival involvement, petechiae, and rapid clinical recovery with vitamin C supplementation were decisive in this case.

**Table 2:** Comparion between scurvy and CRMO CRMO, chronic recurrent multifocal osteomyelitis; DMARD, disease-modifying antirheumatic drug; NSAID, nonsteroidal anti-inflammatory drug

Feature	Scurvy	CRMO
Age at presentation	Elderly and institutionalized individuals or children with restricted diet [1].	Typically, 7 to 12 years [5]
Symptoms	Bone pain, gingival hypertrophy/bleeding, petechiae, edema [2]	Bone pain, swelling, sometimes pustular skin lesions, no gingival involvement [6,8]
Radiology	Subperiosteal hemorrhage, metaphyseal changes, marrow signal abnormalities mimicking osteomyelitis [11,14]	Sterile lytic and sclerotic lesions, symmetrical involvement, vertebral or clavicular lesions [7,8]
Laboratory	Low vitamin C (<1 mg/L) [12]; inflammatory markers often mildly elevated [11]	Inflammatory markers highly elevated; autoimmune workup negative [15]
Response to therapy	Rapid improvement with vitamin C supplementation [4]	Chronic course; managed with NSAIDs, bisphosphonates, or immunosuppressives (methotrexate, sulfasalazine or biologic DMARDs such as etanercept and certolizumab pegol) [8]

This case also teaches valuable clinical lessons. First, a thorough dietary history is indispensable in pediatrics, regardless of socioeconomic status, as selective eating can lead to severe nutritional deficiencies even in children from well-resourced families. Second, scurvy should be considered in the differential diagnosis of multisystem presentations with bone pain and cutaneous symptoms, particularly when all infectious and autoimmune tests are negative. Third, the remarkable improvement after vitamin C treatment emphasizes the importance of addressing dietary deficiencies before undertaking further invasive examinations.

**Figure 5:**
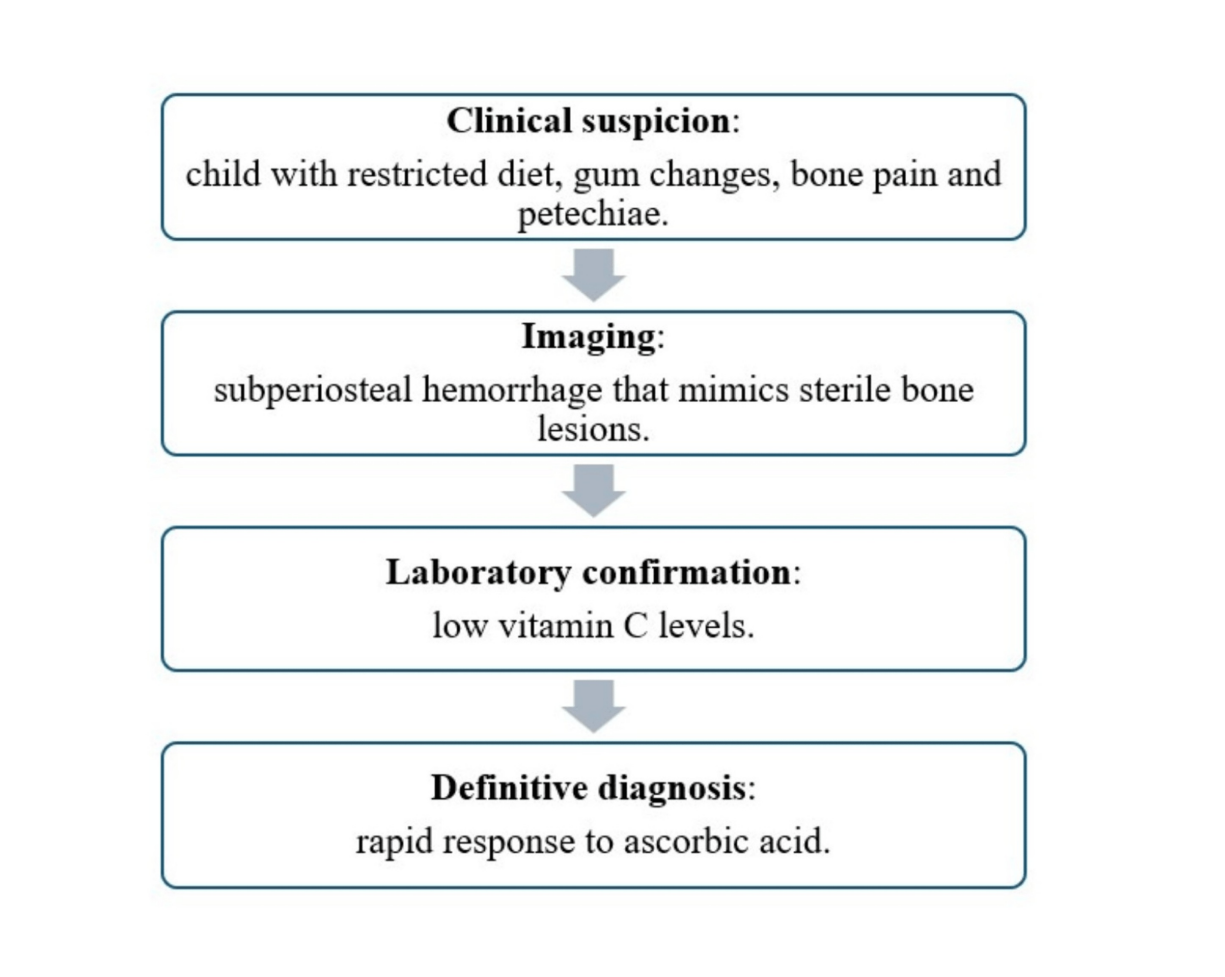
Simplified diagnostic algorithm for pediatric scurvy, highlighting key clinical features, imaging findings, laboratory confirmation, and therapeutic response.

Finally, a simplified diagnostic approach is provided in Figure 5, which may serve as a practical guide.

## Conclusions

Scurvy remains a preventable yet potentially debilitating condition if not promptly recognized. This case underscores the need to maintain clinical suspicion for nutritional deficiencies, even in well-nourished children with no overt socioeconomic risk factors. Our patient’s clinical picture of multifocal bone pain and sterile osteomyelitis on MRI pointed towards CRMO at first. However, his limited diet and severely low vitamin C levels raised scurvy as the true diagnosis, and the rapid therapeutic response confirmed it. Incorporating a thorough dietary history into routine assessments and increasing clinician awareness can prevent costly investigations and unnecessary treatments.
